# Effectiveness of interventions to reduce adverse outcomes among older adults following emergency department discharge: Protocol for an overview of systematic reviews

**DOI:** 10.12688/hrbopenres.13027.2

**Published:** 2021-04-06

**Authors:** Mairéad Conneely, Katie Robinson, Siobhán Leahy, Dominic Trépel, Fionnuala Jordan, Rose Galvin

**Affiliations:** 1 School of Allied Health, Faculty of Education and Health Sciences, Ageing Research Centre, Health Research Institute, University of Limerick, Limerick, Ireland, V94 TPPX, Ireland; 2 Trinity Institute of Neurosciences, School of Medicine, Trinity College Dublin, Dublin, Ireland, DO2 PN40, Ireland; 3 School of Nursing and Midwifery, National University of Ireland Galway, Galway, Ireland, H91 TK33, Ireland

**Keywords:** Older adults, aged, Emergency department, adverse outcomes, interventions, systematic reviews, evidence synthesis, overviews

## Abstract

**Background:** Older adults are frequent users of Emergency departments (ED) and this trend will continue due to population ageing and the associated increase in healthcare needs. Older adults are vulnerable to adverse outcomes following ED discharge. A number of heterogeneous interventions have been developed and implemented to improve clinical outcomes among this cohort. A growing number of systematic reviews have synthesised evidence regarding ED interventions using varying methodologies. This overview aims to synthesise the totality of evidence in order to evaluate the effectiveness of interventions to reduce adverse outcomes in older adults discharged from the ED.

**Methods:** To identify relevant reviews, the following databases will be searched: Cochrane Database of Systematic reviews, Joanna Briggs Institute Database of Systematic Reviews and Implementation Reports, Databases of Abstracts of Reviews of Effects, PubMed, MEDLINE, Epistemonikos, Ageline, Embase, PEDro, Scopus, CINAHL and the PROSPERO register. The search for grey literature will include Open Grey and Grey Literature Reports. Systematic reviews of randomised controlled trials will be analysed to assess the effect of ED interventions on clinical and process outcomes in older adults. Methodological quality of the reviews will be assessed using the Assessment of Multiple Systematic Reviews 2 tool. The review will be reported in accordance to the Preferred Reporting Items for Systematic Reviews and Meta-Analyses statement. Summary of findings will include a hierarchical rank of interventions based on estimates of effects and the quality of evidence.

**Discussion:** This overview is required given the number of systematic reviews published regarding the effectiveness of various ED interventions for older adults at risk of adverse outcomes following discharge from the ED. There is a need to examine the totality of evidence using rigorous analytic techniques to inform best care and potentially develop a hierarchy of treatment options.

**PROSPERO registration**: CRD42020145315 (28/04/2020)

## Introduction

Population ageing is increasing in most countries worldwide ^1^. Across Organisation for Economic Co-operation and Development (OECD) countries, the proportion of the population aged over 65 years has increased from less than 9% in 1960 to 17% in 2015 and is expected to rise to 28% in 2050 ^2, 3^. This change in demographics presents both opportunities and challenges ^4^ Longer life is a valuable resource and presents many opportunities to older adults to have productive and healthy years ^5^. Although increased life expectancy is assumed to be accompanied by an increase in healthy life years, there is little evidence that older adults living today are living with an enhanced health status than their parents did at the equivalent age ^1^. Older adults (aged ≥65 years of age) are the main users of health care services ^6^ and account for a substantial amount of health care costs ^7, 8^.

Multimorbidity (the co-existence of ≥2 chronic conditions) is common in older adults ^1, 9^ and affects more than half of those aged 60 and over ^10, 11^, with increasing prevalence in those aged over 80 years ^1, 4^. Multimorbidity is also correlated with increased health care utilisation and subsequent health care costs ^10^ as multimorbidity can cause problematic clustering of certain morbidities ^12^, and affect treatment of one morbidity and management of another ^1^. The combination of population ageing, multimorbidity and physiological changes in older age ^13^ mean that older adults account for some of the highest percentage of acute care services use ^14^ and have been described as “frequent users” of emergency departments (ED) ^15, 16^, accounting for 12–24% of all Emergency department (ED) attendees ^17, 18^. The reasons why more older adults are seeking ED services are numerous including shortage of aged-care facilities, barriers to accessing primary care services and changes in family demographics ^19^.

Older adults experience longer lengths of stay while in the ED ^18, 20^ and the visits require a high level of urgency and require more resources ^18, 21, 22^. In terms of community support services, international estimates demonstrate that between 45% to 60% of older adults presenting to the ED will be discharged directly home to the community ^23^. A growing body of evidence demonstrates high rates of adverse outcomes post discharge from the ED ^13, 24^ as older adults encounter a period of increased vulnerability following presentation to, and subsequent discharge from, the ED ^15^. A systematic review of 32 prospective and retrospective cohort studies concluded that approximately 20% of older people discharged from the ED return within 30 days, while 17% experience functional decline ^25^. Older adults, who return to the ED early following initial presentation, or index visit, are reported to return for the same complaint again ^18^ indicating concerns that a lack of continuation of appropriate care may contribute to this form of health care utilisation ^24^. There is a high rate of nursing home admission following ED discharge and older adults have a higher rate of mortality than younger age groups following ED discharge ^18, 26^.

The number of adverse outcomes reported following an index visit has led to the development of a number of interventions described in the literature to improve the health status of older adults ^22, 27^. These interventions include single strategies such as ED staffing, modifications strategies to improve ED care delivery such as risk profiling, nurse led interventions, comprehensive geriatric assessments, case management within the ED and post-discharge and discharge planning ^14, 22, 28, 29^. A systematic review of nine studies focusing solely on ED-based interventions reported that interventions that extended beyond referral and those with an integrated model of care (multifaceted interventions) may lead to improved outcomes including nursing home admission, ED revisits, hospitalisation and death ^29^. The authors also reported that the use of a clinical risk screening tool in the ED could potentially allow for identification of older adults most likely to benefit from interventions, but this was not consistent for all outcomes. On the contrary, a systematic review of nine studies by Lowthian *et al.* ^22^ in 2015, reviewed the effectiveness of ED -community transitional strategies such as geriatric assessment, community-based referral, and GP liaison on post-discharge outcomes. This review reported no evidence of the effectiveness of the ED transitional strategy intervention for unplanned revisits, hospitalisation 30 days post discharge or mortality 18 months follow up. A systematic review by Hughes *et al.* (2019) evaluated the effectiveness of ED interventions aimed at improving clinical, patient experience and health care utilisation included 15 studies (9 randomised controlled trials) ^27^. This review explored the impact of interventions that were delivered during the ED visit, following discharge and across the ED-primary care interface using a variety of strategies (case management, discharge planning, and management/medication safety). The authors reported that interventions were heterogeneous with a mixed pattern of effects on clinical and process outcomes.

Given the diverse findings across these systematic reviews, there is a need to conduct an overview of systematic reviews to synthesise the evidence relating to the impact of ED interventions on a number of outcomes for older adults. An overview can highlight gaps in the literature ^30, 31^ and this method of evidence synthesis ^32^ is timely to evaluate the effectiveness of ED interventions on reducing adverse outcomes for older adults following ED discharge. The objectives of this overview are:

1. To identify, appraise and synthesise all relevant systematic reviews of ED based interventions, transitional interventions from the ED to the community and ED initiated interventions to reduce adverse outcomes (clinical outcomes, healthcare utilisation, and patient care experience) in older adults following ED discharge.

2. To identify commonalities and differences between these ED interventions with attention focusing on the characteristics of interventions, the quality of the evidence, the absolute risk difference and other pertinent factors such as heterogeneity (clinical and methodological) within and across reviews.

## Methods

### Protocol

An overview of systematic reviews will be conducted to identify systematic reviews (with/without meta-analysis) investigating the effectiveness of interventions to reduce adverse outcomes in older adults following index visit to the ED. In line with recommendations to improve transparency and reduce potential bias, the authors developed this protocol to outline the key objectives of this overview and what methodology will be employed ^33^. There is an absence of specific reporting guidelines for overviews of reviews of healthcare interventions with the Preferred Reporting Items for Overviews of Reviews (PRIOR) guidelines currently under development ^34^. This protocol was designed in accordance with the methodological framework provided by the Joanna Briggs Institute (JBI) Reviewer’s Manual ^35^, and using the guidance of the relevant items of the Preferred Reporting Items for Systematic Reviews and Meta-Analysis (PRISMA) standardised reporting guidelines ^36^.

This protocol has been prepared with guidance from the PRISMA-Protocols (PRISMA-P) statement ^37^. The PRISMA-P checklist was developed to standardise the conduct and reporting of protocols of systematic reviews that synthesise accumulated data from primary studies, in particular studies that evaluate the effects of interventions and thus not all PRISMA-P items will be applicable for this overview. The relevant sections of the checklist will be used for this protocol in the absence of specific guidelines for the conduction and reporting of overviews of reviews. This methodology has been recommended in the absence of specific guidelines for reporting overviews ^32^. The protocol was registered with PROSPERO on 28th April 2020 (CRD42020145315).

### Search strategy

The authors developed a comprehensive search strategy which has been peer reviewed by a dedicated Education and Health Sciences academic information specialist librarian using the Peer Review of Electronic Searches Model ^38^. The aim of the search strategy is to locate all pertinent research, both published and unpublished systematic reviews, in accordance with best practice for conducting a search strategy for an overview ^35^. A three-step search strategy will be utilised in this overview to ensure a comprehensive search of the literature ^35^. The authors conducted an initial search limited to EMBASE and PubMed databases to identify systematic reviews relevant to the overview research question. Following this, key words within the titles and abstract were identified and analysed and finally index terms for the systematic reviews were analysed in line with the recommendations for conducting a search strategy for an overview ^35^. These steps guided the development of a search strategy including the identified keywords and index terms which will be adapted for each database for the second step of the search strategy ^35^. To illustrate, the full electronic database search strategy for the Embase database is detailed in Table 1.

**Table 1.** Search strategy for Embase Database.

NUMBER	QUERY	RESULTS
1	'aged'/exp OR 'aged	4,503,863
2	'older adults':ti,ab OR 'older adult':ti,ab OR 'older people':ti,ab OR 'older patient':ab,ti OR 'older patients':ab,ti OR 'very elderly' OR senior:ti,ab OR seniors:ab,ti OR 'aged':ti,ab OR 'geriatric patient':ab,ti OR 'geriatric care'/exp OR 'geriatric care' OR 'geriatrics'/exp OR 'geriatrics' OR geriatric:ti,ab OR 'geriatric assessment'/exp OR 'geriatric assessment' OR 'elderly care':de OR 'gerontology':ab,ti	1,276,936
3	'very elderly'/exp OR 'very elderly'	191,476
4	'emergency health service'/exp OR 'emergency health service'	100,904
5	'emergency department':ab,ti OR 'emergency departments':ab,ti OR 'emergency ward':ab,ti OR'emergency treatment':ab,ti OR 'emergency health service' OR 'emergency room':ab,ti OR 'hospital':ab,ti OR 'emergency unit':ab,ti OR 'trauma unit':ab,ti OR 'emergency nursing':ab,ti OR'emergency care':ti,ab OR 'acute medical unit':ab,ti OR 'emergency medicine':ab,ti	1,695,708
6	'systematic review':ab,ti	184,484
7	#1 OR #2 OR #3	4,676,994
8	#4 OR #5	1,696,511
9	#7 AND #8	497,954
10	#6 AND #9	355

The third step will involve a manual search for systematic reviews via a search of the reference lists of all included systematic reviews selected for critical appraisal ^35^.

To identify relevant systematic reviews, the following electronic databases will be searched following recommendations from the JBI Reviewers Manual ^35^: the Cochrane Database of Systematic Reviews, Joanna Briggs Institute Database of Systematic Reviews and Implementation Reports, Databases of Abstracts of Reviews of Effects, PubMed, 1966 to date; OVID Medline, 1996 to date; Embase, 1974 to date; Cumulative Index to Nursing and Allied Health Literature (CINAHL) (EBSCO Host), 1981 to date; Epistemonikos; AGELINE, 1978 to date; PEDro, 1999 to date; Scopus and the PROSPERO register ^39^. A comprehensive search will encompass a search of the grey literature, reports from governments and non-government organisations as per best practice in conducting an overview ^35, 39^.

### Study selection

***Screening.*** A two-stage process will be utilised to examine the results of the search strategies of all databases. Citations from each database will be exported by MC to a master reference management library, EndnoteX8 (Clarivate Analytics, PA, USA) and duplicates will be removed by MC. Stage 1 will involve screening of titles and abstracts in this master database by two independent reviewers (MC and RG) against the inclusion criteria for the overview as per best practice ^35^. In Stage 2, full text articles will be retrieved for all systematic reviews that meet the inclusion criteria for the overview identified in the initial screening (Stage 1) and also for studies where there is a query on based on the Stage 1 screening of title and abstract.

A comparison of these systematic reviews will be conducted by the same two independent reviewers (MC and RG) and discrepancies will be resolved by consensus or by a third reviewer (SL). The process of the entire search and selection processes will be presented in a PRISMA flow diagram.

### Inclusion and exclusion criteria

***Types of studies.*** The unit for analysis will be quantitative systematic reviews with or without meta-analysis, and research synthesis that investigates the effectiveness of ED interventions delivered to older adults following discharge from the ED. Eligible systematic reviews will be appraised by two independent reviewers (MC and RG) for methodological quality prior to inclusion in the overview, using a standardised critical appraisal tool, JBI Critical Appraisal Checklist for Systematic Reviews and Research Synthesis ^35^. Any disagreements that arise between the two reviewers will be resolved through consensus or discussion or guidance from a third reviewer (SL) will be employed ^35^. A narrative summary of the results of the critical appraisal of systematic reviews will be presented supported by relevant supporting tables and/or figures ^35^. Following discussion between authors, the quality of each systematic review will be based on the predetermined criteria ^35, 40, 41^.

A score of 0–3 representing very low-quality score; a score of 4–6 representing a low quality score; a score of 7–9 representing a moderate-quality score; and a score of 10–11 will be considered a high-quality score. A score of 0-3 indicates a very low-quality systematic review, and thus a systematic review will be excluded if it does not meet >3 of the 11 criteria ^35^.

This overview of systematic reviews will include systematic reviews published in any language. If a systematic review is an update of a previous systematic review, the most recent and highest quality systematic review will be considered and the lower quality systematic review will be excluded from the overview ^39^.

### Eligibility criteria using PICOT framework

***Population.*** This overview will consider existing systematic reviews that include older adults (65 years and over) following an index visit to the ED or Acute Medical Unit (AMU) discharged within 72 hours of index visit.

***Interventions.*** Systematic reviews that analyse the effect of ED based interventions, transitional interventions and ED initiated interventions on outcomes for older adults who present to the ED with an index complaint.

**Comparator:**

All comparators will be considered.

### Outcomes:

***Primary clinical outcome.*** Functional status/decline

- Systematic reviews reporting overall functional status including measures of functional ability assessed using a validated tool such as:

A measure of functional decline or ability (Activities of Daily Living):

Barthel’s ADL Index (BI),

Functional Independence Measure (FIM),

Physical functioning aspect of the Health Related Quality of Life Short Form 36

***Secondary outcomes***

Secondary Clinical outcomes

- Health related Quality of life (EuroQol, EQ-5D)
- Mortality

Secondary outcomes

- Healthcare Utilisation: ED readmission, hospital admission rates (following ED discharge)
- Patient experience or satisfaction: studies reporting any validated measure of patient experience and satisfaction
- ED Length of stay (LOS)

Table 2 summarises the population, intervention, comparator, outcome and study design (PICOS) statement.

**Table 2.** PICOTS Statement.

STUDY CHARACTERISTIC	INCLUSION CRITERIA
Population	Systematic reviews including older adults aged 65 years and over who present to an ED for acute, urgent or emergency care.
Interventions	Any intervention strategy including: Comprehensive geriatric assessment within the ED Geriatric nursing assessment within the ED Interventions initiated in the ED used to guide appropriate follow-up and referral Discharge Planning Case Management Medication safety Strategies guided by 2014 Geriatric Emergency Department guidelines
Comparator	Systematic reviews that include studies that compare interventions to usual or enhanced care (e.g. information or educational control)
Outcomes	• Primary outcomes: Functional decline Overall functional status (or sub domains of physical or mental functioning), • Secondary outcomes : health related quality of life; mortality; Patient satisfaction/experience (any validated measure of patient satisfaction/experience); Healthcare utilisation: ED readmission; unplanned hospital admission (following ED discharge)
Setting	Emergency departments
Study design	Quantitative systematic reviews of randomised controlled trials with or without meta-analysis
Timing	Time points that are logically affected by the intervention and are clinically relevant, including short (e.g. 30 days) and longer (e.g. 90 days) time points

### Public and patient involvement

Members of the public and patients will not be involved in this overview of systematic reviews. The authors anticipate that the findings of this review (which represents Phase 1 of the Medical Research Council framework for developing and evaluating complex interventions ^42^) will represent the first stage in the design of a pilot intervention to address the risk of adverse outcomes in older adults following discharge from the ED. The subsequent phases will have a strong public and patient involvement.

***Data collection and extraction.*** Two independent reviewers (MC and RG) will extract data from the selected systematic reviews using the standardised data extraction tool in JBI SUMARI ^39^. This will be piloted to ensure that the content and mechanism of data recording is accurate. The following information will be extracted from each systematic review as recommended by the JBI Manual for the conduct of overviews ^35^:

1. Citation details (authors and year of publication)
2. Objectives of the included systematic review
3. Type of review
4. Study population
5. Setting and context
6. Number of databases searched
7. Date range of database searching
8. Publication date range of studies included in the review that inform each outcome of interest
9. Number of randomised controlled trials (RCTs) included and the country of origin of the RCT
10. Tool used to critically appraise the primary studies and their quality rating
11. Outcomes reported that are relevant to the overview research question with effect estimates, SE and CI as available.
12. Methods of analysis employed to synthesis the evidence
13. Comments of overview authors regarding any included study, including potential confounding variables

Should any disagreements arise between the two reviewers, these will be resolved through discussion or with guidance from a third reviewer (SL) ^35^. Should a systematic review present unclear, missing or incompletely reported data, we will endeavour to contact the authors of the systematic review to obtain the data and document same.

## Methodological quality of included reviews

The methodological quality of the included systematic reviews will be assessed by two independent reviewers (MC and RG) using the Assessment of Multiple Systematic Reviews 2 (an update of AMSTAR) tool ^43^. The AMSTAR 2 is a 16-item checklist utilised to assess the quality of systematic reviews that include randomised or non-randomised studies of healthcare interventions ^43^. The AMSTAR-2 includes 10 items from the original AMSTAR tool ^44^. Reviewers score each domain with ‘yes’ or ‘no’, or in some domains there is a third option of ‘partial yes’. The quality of each systematic review will be rated as high, moderate, low and critically low. Any disagreements that may arise will be resolved through discussion or will be addressed by a third reviewer (SL).

### Assessing the quality of evidence

An algorithm that assigns the Grading of Recommendations, Assessment, Development and Evaluation (GRADE) ^30, 45, 46^ framework level of evidence will be used to grade the certainty of evidence. This algorithm is a new methodological approach to assessing the quality and certainty of evidence in overviews ^46^ and has been used in recent overviews ^47, 48^. This approach will assess the quality of the evidence relating to the primary and secondary outcomes included in RCTs in systematic reviews as detailed above. Two independent reviewers (MC and RG) will assess the quality of evidence for each outcome in each systematic review independently. Any disagreements that may arise will be resolved through discussion or will be addressed by a third reviewer (SL). In this algorithm, each systematic review starts with a ranking of high certainty (no downgrade) and is downgraded one level per serious methodological concerns as outlined in Box 1 below.

**Box 1. Algorithm for applying GRADE level of evidence in systematic reviews ^48^**

**A systematic review is downgraded 1 Level as per the following methodological concerns:**

1. Number of participants within pooled analyses (100–199 participants)

2. Risk of bias in randomisation and blinding for <75% included studies

3: Heterogeneity as measured by a recognised measure of statistical heterogeneity, *I²* > than 75%;

4: ‘No’ to one of the AMSTAR 2 questions 2, 4, 5 and 6 (corresponding to a priori research design, search characteristics, independence of study design and data extraction).

**A systematic review is downgraded two levels per very serious methodological concerns:**

1: Number of participants within pooled analyses (1–99 participants)

2: ‘No’ to two or more of the AMSTAR 2 questions 2, 4, 5 and 6 (corresponding to a priori research design, search characteristics, independence of study design and data extraction).

***Dealing with overlap.*** The issue of overlapping reviews (studies appearing in more than one review) is a challenge to authors of overviews. A matrix of evidence table will be collated and examined by two independent reviewers (MC and RG) to assess the amount of overlap between systematic reviews. Should multiple systematic reviews exist investigating the population for the same outcome, the following will be applied:

1. If the primary studies are completely overlapping, the most recent, highest quality (based on the AMSTAR 2), most relevant, and most comprehensive systematic review will be selected.
2. If the primary studies partially overlap, both reviews will be retained if the lower quality review consists of more than one-third new studies.

### Data synthesis and analysis

The results extracted from each systematic reviews will be presented both quantitatively and qualitatively to answer the objectives of this overview ^35^. The authors will present key quantitative results in tables accompanied by narrative interpretation as per best practice in presenting a summary of evidence in an overview ^39^. The results of the various sections of the overview will be presented in a Summary of Evidence table that will name the ED intervention (s), identify the systematic review(s) and provide a clear indication of the results ^39^. Given the anticipated heterogeneity, the findings will be summarised using a narrative synthesis approach.

The data contained within each systematic review (including effect estimates and 95% confidence intervals) will be reported in a narrative summary. Interventions will be ranked according to estimates of the absolute risk difference and the results of the methodological quality of the evidence ^35^. A summary of ED interventions will be developed with consideration of the certainty of the evidence and AMSTAR-2.

## Discussion

This overview will employ robust methodology to present a synthesis of evidence from systematic reviews regarding the effectiveness of ED interventions and strategies on reducing adverse outcomes in older adults following index visit to the ED. Given the breadth of interventions and the diversity of the findings reported in systematic reviews, there is a need to conduct an overview to provide a broader and high-quality evidence synthesis. This overview will identify systematic reviews, and compare and contrast the results of several systematic reviews, as well as explore the reasons for the findings. As overviews are a new form of research synthesis, a number of challenges regarding the methodological conduct of an overview are described in the literature ^33, 49, 50^. These issues will be discussed when presenting the findings of the overview. To the best of our knowledge this is the first overview of systematic reviews published exploring this research question.

## Dissemination of findings

The findings of this umbrella review will be disseminated through the publication of peer-reviewed manuscripts. Additionally, findings will be presented at both national and international conferences and via a Public and Patient Involvement group of older adults.

## Study status

Searching and screening have been completed.

## Data availability

### Underlying data

No data are associated with this article.

### Reporting guidelines

Figshare: PRISMA-P checklist for ‘Effectiveness of interventions to reduce adverse outcomes among older adults following emergency department discharge: Protocol for an overview of systematic reviews’ https://doi.org/10.6084/m9.figshare.12179022.v1 ^51^

Data are available under the terms of the Creative Commons Attribution 4.0 International license (CC-BY 4.0).
