# Supplementary material for: Effectiveness of interventions to reduce adverse outcomes among older adults following emergency department discharge: Protocol for an overview of systematic reviews
Source: HRB Open Res. 2021 Apr 6;3:27. Originally published 2020 May 22. [Version 2] doi: 10.12688/hrbopenres.13027.2 (PMC8078215; doi:10.12688/hrbopenres.13027.2)
Supplement: Peer review history

# Peer review: Reviewer response for version 2

**DOI:** 10.21956/hrbopenres.14436.r29251

**Published:** 2021-05-24

**Authors:** Ruth McCullagh

**Affiliations:** 1 Discipline of Physiotherapy, School of Clinical Therapies, University College Cork, Cork, Ireland

Thank you very much for the opportunity to review your protocol. I have no other suggestions to make and wish you the very best of luck with the project.

Is the study design appropriate for the research question?

Yes

Is the rationale for, and objectives of, the study clearly described?

Yes

Are sufficient details of the methods provided to allow replication by others?

Yes

Are the datasets clearly presented in a useable and accessible format?

Not applicable

Reviewer Expertise:

Older adult rehabilitation and physiiotherapy

I confirm that I have read this submission and believe that I have an appropriate level of expertise to confirm that it is of an acceptable scientific standard.

# Peer review: Reviewer response for version 2

**DOI:** 10.21956/hrbopenres.14436.r29252

**Published:** 2021-04-26

**Authors:** Michelle Gates

**Affiliations:** 1 Alberta Research Centre for Health Evidence, Department of Pediatrics, University of Alberta, Edmonton, AB, Canada

Thank you for considering my earlier suggestions.

 Abstract:

- Good overall. I would still strongly recommend not hierarchically ranking the interventions (other than potentially by strength of evidence). I think you have removed this from the text, so it would be good to remove here as well.

Methods:

- Please note that PRISMA is a reporting guideline, not a methodological standard. Therefore, it would be preferable to say that you will report the review according to PRISMA. I would say the same for PRISMA-P, it should not guide conduct, but reporting only. For conduct you may e.g., follow the Cochrane Handbook or other methodological guidance.
- I would say that GRADE is not a new approach, but indeed it has not yet been adapted for use in overviews. It therefore is useful that you have provided an algorithm.
- For GRADE, often 300 events is considered adequate for precision. For risk of bias, you may want to check how this impacts the results. Heterogeneity may also encompass the direction of effects that you see (and not just the I2).
- Note that a poor quality systematic review may still report data from good studies. This might be something to think about when applying GRADE based on AMSTAR score.

Is the study design appropriate for the research question?

Yes

Is the rationale for, and objectives of, the study clearly described?

Partly

Are sufficient details of the methods provided to allow replication by others?

Partly

Are the datasets clearly presented in a useable and accessible format?

Not applicable

Reviewer Expertise:

Evidence synthesis

I confirm that I have read this submission and believe that I have an appropriate level of expertise to confirm that it is of an acceptable scientific standard.

# Peer review: Reviewer response for version 1

**DOI:** 10.21956/hrbopenres.14122.r28503

**Published:** 2021-03-15

**Authors:** Michelle Gates

**Affiliations:** 1 Alberta Research Centre for Health Evidence, Department of Pediatrics, University of Alberta, Edmonton, AB, Canada

Thank you for the opportunity to review this protocol for an overview of reviews of interventions in the emergency department to reduce adverse outcomes among older adults following discharge (e.g., return to ED, among others). It seems generally well planned. I have listed some points below that could be addressed to improve the planned overview.

 Introduction:

- Before 'longer life is a valuable' it looks like a new sentence should start.
- Could be more specific in the objective statement about which PICOTs are of interest (e.g., which interventions, in specific population, and specific outcomes).
- Can likely remove the paragraph describing overviews of reviews. Would then replace the earlier objective statement with one or all of the 3 specific objectives.
- I think that identification of the evidence is part of the overview process but should not be a specific objective.
- It would be good for the objectives to be in PICOT format.

Methods:

- Please note that the PRISMA and PRISMA-P are reporting guidelines, they are not meant to guide design.
- For the search I think it might be useful to include terms other than 'systematic review' as these are often named differently (e.g., review, meta-analysis, among others).
- It is not fully clear from the description how full text screening will happen. Please clarify.
- Why will pooled analyses (I am assuming this is from non-systematic reviews) be included?
- Are only high quality systematic reviews going to be included? It is not clear. If not, I am not sure why critical appraisal is happening at the selection stage.
- In the eligibility criteria, please indicate comparators of interest in the text.
- Are you including only reviews of RCTs? It seems like this from the data extraction section but I do not see this within the eligibility criteria.
- It is not clear why the quality of the systematic reviews is being assessed twice - first the JBI criteria are listed at the selection stage, then later AMSTAR-2.
- Dealing with overlap - if both reviews are kept, what will you do about the overlap? What will you do if the reviews appraised their primary studies differently?
- What will you do if reviews do not report an appraisal of the quality of their included studies? Will you appraise these yourself, or report them as missing?
- What will you do if all the information needed for GRADE is not available within the systematic reviews? For e.g. if quality appraisal is missing as above?
- Would it make sense (if not conducting new syntheses) to extract GRADE assessments directly from the systematic reviews?
- It looks like the evidence will be presented qualitatively only. Will there be meta-analysis? If not, I would not say there will be a quantitative synthesis.
- I would caution strongly against a hierarchical summary of results, as it is important to not make informal indirect comparisons across the findings of different systematic reviews. Please see Cochrane handbook Ch 5 for more information on this. It is difficult to assess the transitivity assumption in overviews of reviews.

Is the study design appropriate for the research question?

Yes

Is the rationale for, and objectives of, the study clearly described?

Partly

Are sufficient details of the methods provided to allow replication by others?

Partly

Are the datasets clearly presented in a useable and accessible format?

Not applicable

Reviewer Expertise:

Evidence synthesis

I confirm that I have read this submission and believe that I have an appropriate level of expertise to confirm that it is of an acceptable scientific standard, however I have significant reservations, as outlined above.

# Response

**Published:** 2021-03-22

**Authors:** Mairead Conneely

**Affiliations:** University of Limerick, Ireland

Many thanks for your time spent reviewing this protocol and for your constructive and insightful feedback and comments. We have reflected upon your feedback and made revisions to our manuscript in line with it. Please see below a detailed point by point response to all comments (reviewer's comments in **bold** and authors’ responses in black).

**SECTION: INTRODUCTION**

**Comment 1:** **Before 'longer life is a valuable' it looks like a new sentence should start.**

 Response: Many thanks for this comment. This error has been amended

**Comment 2: Could be more specific in the objective statement about which PICOTs are of interest (e.g., which interventions, in specific population, and specific outcomes).**

 Response: Many thanks for this comment. The objectives have been edited for more explicit statement aligned to PICOT framework.

**Comment 3: Can likely remove the paragraph describing overviews of reviews. Would then replace the earlier objective statement with one or all of the 3 specific objectives**

 Response **:  **Many thanks for this comment. This section has been condensed to one sentence to link the previous paragraph to the objectives of the Overview.

**Comment 4: I think that identification of the evidence is part of the overview process but should not be a specific objective.**

 Response: Many thanks for this comment. We have merged objectives one and 3 in the revised manuscript.

**Comment 5: It would be good for the objectives to be in PICOT format**

 Response: Many thanks for this comment. The objectives have been edited to specify the PICOT statement.

**SECTION: METHODS**

****

**Comment 1:Please note that the PRISMA and PRISMA-P are reporting guidelines, they are not meant to guide design.**

 Response: Many thanks for this comment. The wording has been edited for clarity.  

**Comment 2:** **For the search I think it might be useful to include terms other than 'systematic review' as these are often named differently (e.g., review, meta-analysis, among others).**

 Response:  Many thanks for this comment. The JBI Manual of Evidence Synthesis Chapter 10 recommends the use of the term “systematic review” to broaden the search as far as possible and recommend not using terms such as meta-analysis as the return may be limited to meta-analysis and risk losing out of potentially relevant systematic reviews.   We sought clarification on this also from the information specialist and she concurred with the process outlined by JBI.

**Comment 3: It is not fully clear from the description how full text screening will happen. Please clarify.**

 Response: ****Many thanks for this comment. Further details have been added to this section for an explicit description of the screening process.

**Comment 4: Why will pooled analyses (I am assuming this is from non-systematic reviews) be included**

 Response: Many thanks for this comment. The JBI Manual recommends including pooled analyses due to different terms used. For comprehensiveness, if there was a systematic review that did pooled analysis, that met our PICOT, it would be pertinent to include. However, we understand the confusion with different terminology and have deleted. 

**Comment 5:  Are only high quality systematic reviews going to be included? It is not clear. If not, I am not sure why critical appraisal is happening at the selection stage**

 Response **: **Many thanks for this comment. We have amended the updated manuscript to address this query. All relevant systematic reviews will be screened for inclusion using the JBI Critical Appraisal Checklist for Systematic Reviews and Research Synthesis. A score of 0-3 indicates a very low-quality score and such studies will be excluded. This initial screening of quality using the JBI Checklist has been conducted in other overviews also and we have referenced same in the manuscript.

**Comment 6: In the eligibility criteria, please indicate comparators of interest in the text.**

 Response: Many thanks for this comment. Comparators have been added to the text as part of the PICOT.

**Comment 7: are you including only reviews of RCTs? It seems like this from the data extraction section but I do not see this within the eligibility criteria.**

 Response: Many thanks for this comment. This is already outlined in the PICOT statement in Table 2. 

**Comment 8: It is not clear why the quality of the systematic reviews is being assessed twice - first the JBI criteria are listed at the selection stage, then later AMSTAR-2.**

 Response: Many thanks for this comment.  The sentence on the scoring of the JBI Critical Appraisal has been edited to make explicit the exclusion of a systematic review based on >3 of the 11 criteria as suggested in the JBI Manual for the conduct of systematic reviews. Thus only Systematic reviews that score higher than 3/11 will be included in this overview. Thus the JBI Critical appraisal tool will be  used as a screening tool for a minimal acceptable standard for a systematic review. The methodological quality of the included systematic reviews will be assessed using the AMSTAR 2 tool. The use of the AMSTAR 2 tool always informs the algorithm to GRADE for evaluation of the quality of evidence.

**Comment 9: Dealing with overlap - if both reviews are kept, what will you do about the overlap? What will you do if the reviews appraised their primary studies differently?**

 Response: Many thanks for this comment. In the presence of complete overlap between reviews, the highest quality review, as determined by the AMSTAR 2, will be included in data synthesis and analysis. In cases, where there is complete overlap and the reviews receive the same rating using the AMSTAR 2, then the most recently published review will be included. However, for this umbrella review our unit of analysis is the systematic review and consequently, we will not be performing a quality rating of the primary studies. This is described in Methods section, subsection "Dealing with Overlap" of the manuscript.

**Comment 10: What will you do if reviews do not report an appraisal of the quality of their included studies? Will you appraise these yourself, or report them as missing?**

 Response: Many thanks for this comment. We agree that the use of a predetermined tool to assess the quality rating of primary studies in included systematic reviews can strengthen the review. However, for this overview review our unit of analysis is the systematic review and consequently, we will not be performing a quality rating of the primary studies. As part of the JBI Critical Appraisal Checklist for Systematic Reviews and Research Syntheses, we will record and present whether systematic reviews completed critical appraisal by two or more reviewers independently. Additionally, as part of the AMSTAR 2 we will be recording if the authors of the systematic reviews used a satisfactory tool to assess for risk of bias.

**Comment 11: What will you do if all the information needed for GRADE is not available within the systematic reviews? For e.g. if quality appraisal is missing as above**

 Response: Many thanks for this comment. As per the recommendations outlined by Pollock et al 2017, which we have referenced,   “If ROB for individual trials was not reported within the review, we

 were conservative and assumed that less than 75% of participants had low ROB”. We have added a box detailing the algorithm to GRADE for further details on the algorithm. 

**Comment 12: Would it make sense (if not conducting new syntheses) to extract GRADE assessments directly from the systematic reviews?**

 Response: Many thanks for this comment. Cochrane systematic reviews will have this Grade Assessment detail. However, non-Cochrane reviews will may not present an assessment of the quality of the evidence or may present many methods. For consistency, we will apply the GRADE algorithm to all systematic reviews as opposed to extracting the regular GRADE which would be a different tool.

 The unit of analysis is a systematic review and the algorithm to GRADE is applied at the level of the systematic review.

**Comment 13: It looks like the evidence will be presented qualitatively only. Will there be meta-analysis? If not, I would not say there will be a quantitative synthesis.**

 Response: Many thanks for this comment. The wording has been edited to highlight qualitative only. 

**Comment 14:  I would caution strongly against a hierarchical summary of results, as it is important to not make informal indirect comparisons across the findings of different systematic reviews. Please see Cochrane handbook Ch 5 for more information on this. It is difficult to assess the transitivity assumption in overviews of reviews.**

 Response: Many thanks for this comment. The JBI Manual recommends the use of a hierarchical summary of results. However, we understand the reservation with the use of the term and have edited this section accordingly.

# Peer review: Reviewer response for version 1

**DOI:** 10.21956/hrbopenres.14122.r27616

**Published:** 2020-07-10

**Authors:** Ruth McCullagh

**Affiliations:** 1 Discipline of Physiotherapy, School of Clinical Therapies, University College Cork, Cork, Ireland

Thank you for giving me the opportunity to review your protocol. 

 The abstract reports need for the study and the planned methods clearly. 

 The background explains clearly the conflicting outcomes of trials and existing systematic reviews, highlighting the complexity of the problem. An overview of systematic reviews is clearly justified.

 The methods described are aligned with the recommended methods and show sound methodology, with a comprehensive search strategy, citeris that match the research question, methods to limit their bias and evaluation of methodological quality of the data. 

 Wishing you success and I ​​​look forward to reading the outcome of the study.

Is the study design appropriate for the research question?

Yes

Is the rationale for, and objectives of, the study clearly described?

Yes

Are sufficient details of the methods provided to allow replication by others?

Yes

Are the datasets clearly presented in a useable and accessible format?

Not applicable

Reviewer Expertise:

Older adult rehabilitation and physiiotherapy

I confirm that I have read this submission and believe that I have an appropriate level of expertise to confirm that it is of an acceptable scientific standard.

# Response

**Published:** 2020-07-10

**Authors:** Mairead Conneely

**Affiliations:** University of Limerick, Ireland

Many thanks for reviewing our protocol Dr McCullagh

 Mairéad Conneely
